# Functional Subunits of Eukaryotic Chaperonin CCT/TRiC in Protein Folding

**DOI:** 10.4061/2011/843206

**Published:** 2011-07-02

**Authors:** M. Anaul Kabir, Wasim Uddin, Aswathy Narayanan, Praveen Kumar Reddy, M. Aman Jairajpuri, Fred Sherman, Zulfiqar Ahmad

**Affiliations:** ^1^Molecular Genetics Laboratory, School of Biotechnology, National Institute of Technology Calicut, Kerala 673601, India; ^2^Department of Biosciences, Jamia Millia Islamia, Jamia Nagar, New Delhi 110025, India; ^3^Department of Biochemistry and Biophysics, University of Rochester Medical Center, NY 14642, USA; ^4^Department of Biology, Alabama A&M University, Normal, AL 35762, USA

## Abstract

Molecular chaperones are a class of proteins responsible for proper folding of a large number of polypeptides in both prokaryotic and eukaryotic cells. Newly synthesized polypeptides are prone to nonspecific interactions, and many of them make toxic aggregates in absence of chaperones. The eukaryotic chaperonin CCT is a large, multisubunit, cylindrical structure having two identical rings stacked back to back. Each ring is composed of eight different but similar subunits and each subunit has three distinct domains. CCT assists folding of actin, tubulin, and numerous other cellular proteins in an ATP-dependent manner. The catalytic cooperativity of ATP binding/hydrolysis in CCT occurs in a sequential manner different from concerted cooperativity as shown for GroEL. Unlike GroEL, CCT does not have GroES-like cofactor, rather it has a built-in lid structure responsible for closing the central cavity. The CCT complex recognizes its substrates through diverse mechanisms involving hydrophobic or electrostatic interactions. Upstream factors like Hsp70 and Hsp90 also work in a concerted manner to transfer the substrate to CCT. Moreover, prefoldin, phosducin-like proteins, and Bag3 protein interact with CCT and modulate its function for the fine-tuning of protein folding process. Any misregulation of protein folding process leads to the formation of misfolded proteins or toxic aggregates which are linked to multiple pathological disorders.

## 1. Introduction

The primary amino acid sequence of a protein contains all the information necessary for protein folding and its biological activity [1]. However, in a normal cellular condition, a nascent polypeptide chain faces a crowded environment and there is a good possibility that protein will be misfolded and will form aggregates that make the protein inactive, and in certain cases it becomes toxic for the cell. Both the prokaryotic and eukaryotic cells possess a family of proteins responsible for binding to nascent polypeptide chains and help them fold into biologically functional three-dimensional structures, they are known as molecular chaperones, and they vary in size and complexity [2–6]. Many of the molecular chaperones are induced in response to stress or heat, and so they got the name Hsp (heat shock protein). Molecular chaperones like Hsp90, Hsp70, Hsp40, and Hsp104 bind to nascent polypeptide chain at hydrophobic regions which are exposed to the crowded environment otherwise buried inside in a completely folded protein [7–10]. Molecular chaperones have developed multiple and diverse tertiary and quaternary structures to bind nonnative protein substrates. Though, there is a lack of sequence similarity among different families of chaperones and only a few of them are represented in all three domains of life (bacteria, archaea, and eukaryote), generally, they use convergent strategies to bind the substrates. Crystallographic and other evidence show that many chaperones including prefoldin, trigger factor, hsp40, and hsp90 have clamp-like structures, possibly responsible for the binding of nonnative substrates [11]. Another class of cylindrical-shaped chaperones, known as chaperonins, is found to be conserved in all three domains of life and assist the folding of many cytosolic proteins [12, 13]. In some cases, the transient binding of nascent polypeptide chain is sufficient for protecting its hydrophobic regions and promoting its proper folding. However, for the folding of a multidomain protein, more than one class of chaperones might be involved, and they work in a concerted manner to generate a protective passage. For example, nascent polypeptide chain coming out from ribosome will first bind to Hsp70/Hsp90 which will help attain a quasinative structure and then will be transferred to chaperonin CCT for its final folding [14, 15]. Here we review the current status of understanding of protein folding by the chaperonin CCT complex in eukaryotes.

The chaperonins are large, multimeric, cylindrical protein complexes consisting of two stacked rings and each ring has 7–9 subunits [2, 4, 16–18]. On the basis of amino acid sequence homology, chaperonins have been categorized into two groups, group I and group II [19–24]. Group I is found in all eubacteria and endosymbiotic organelles like mitochondria, chloroplasts, and related organelles like hydrogenosomes and mitosomes whereas group II chaperonins are present in archaebacteria and in the cytosol of all eukaryotes [2, 17, 25–27]. Here we will give brief introduction to group I chaperonin and then will discuss group II chaperonin, CCT.

## 2. Group I Chaperonin

The function of this group has been well studied using GroEL/GroES from *Escherichia coli*. The genes for GroEL and GroES were discovered in a mutagenic screen for genes required for the growth of bacteriophage lambda and later found to be essential for the survival of *E. coli* itself in all conditions [28–30]. GroEL/GroES has been the subject of extensive structural and functional analysis for understanding protein folding *in vitro* and *in vivo* [31–33]. GroEL is about 800 kDa homooligomeric protein complex with ATPase activity and is composed of two seven-membered rings of 57 kDa subunits. Each ring has a central cavity aligned with hydrophobic surfaces for the binding of unfolded or denatured proteins [34–37]. The cochaperonin GroES is a heptameric ring complex composed of 10 kDa subunits and caps the GroEL folding chamber [38–40]. The folding cage generated by GroEL/GroES plays dual functions in protein folding. First, confinement of substrate protein in the GroEL/GroES complex is necessary for protecting them from aggregation. Second, the folding process in the chaperonin would be much faster than that of in a free solution [41]. X-ray studies of GroEL have revealed three distinct domains: equatorial domain, apical domain, and intermediate domain [42]. The equatorial domain is responsible for most of the intra- and intersubunit interactions as well as for the binding of ATP and its hydrolysis. The apical domain encompasses the entrance of central cavity and holds all the hydrophobic residues required for substrate binding. The intermediate domain connects both the domains and acts as a hinge for the movements of apical domain which is induced upon binding of ATP and its hydrolysis [43–45]. The substrate binding residues present in the apical domain is also responsible for binding of co-chaperonin, GroES, which is essential for GroEL-mediated protein folding. The mechanism of GroEL-mediated protein folding has been extensively studied using different approaches [16, 24, 46, 47]. Briefly, protein folding cycle starts with the binding of unfolded or denatured proteins and ATP at one end (the cis end) of GroEL, followed by the binding of GroES to the same end. The binding of GroES caps the entrance and releases the substrate to the central cavity. The binding of GroES also promotes ATP hydrolysis and the protein substrate gets folded in the “Anfinsen cage” of the GroEL-GroES-ADP complex. The binding of protein substrate and ATP to trans ring causes the release of GroES and substrate [48].

## 3. Group II Chaperonin

In the group II chaperonins, both archaeal thermosome and eukaryotic chaperonin containing TCP-1 (CCT; also known as TCP-1 ring complex, TRiC) are being studied using different techniques [49–52]. However, here we will confine our discussion to CCT complex only. The subunit TCP-1 of CCT complex was first isolated from murine testes and subsequently it was found to be constitutively expressed in other mammalian cells, insects, and yeasts [53–57]. The compelling evidence for CCT complex as a chaperonin came from the observations of its involvement in the assembly of actin and tubulin filaments [58–61]. Though, initially, it was discovered as a folding machine for actin and tubulin, later, it was found to be involved in the folding of 5–10% of newly synthesized cytosolic protein substrates [12, 52, 62, 63]. Like GroEL system, CCT also binds ATP and hydrolyses it during protein folding cycle, the CCT does not have detachable GroES-like cochaperonin, rather, a flexible protrusions located in the apical domain in each CCT subunit acts as a lid and is responsible for closing the central cavity [17, 64, 65].

### 3.1. Structure and Ring Arrangement of CCT

**Figure 1:**
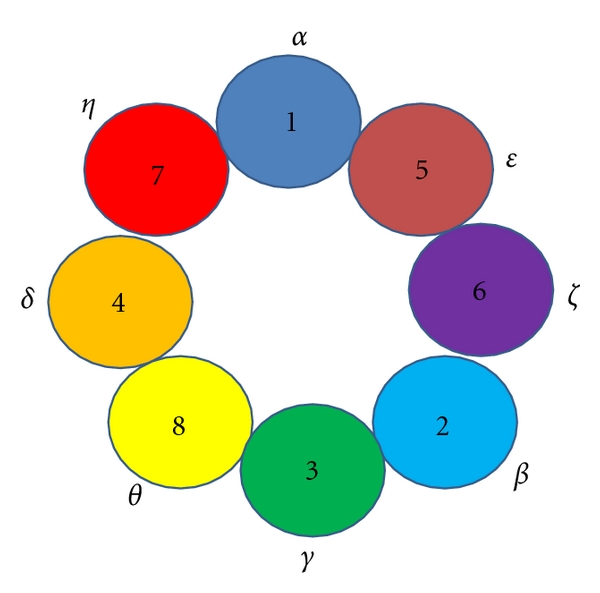
The intra-ring subunit arrangement of CCT. Both Greek alphabets and Arabic numbering system have been used to denote each of the subunits (this was made on the basis of [71]).

The chaperonin CCT is a large, cylindrical, multimeric complex having central cavity for binding unfolded or denatured polypeptides. The structure of CCT is much more complex compared to GroEL because each ring of CCT is composed of eight different but related subunits ranging between 52 and 65 kDa [58–60]. Moreover, it has been observed that all the eight subunits of CCT are essential in yeast [52]. Now the question is when homooligomeric GroEL is sufficient to fold an array of substrates, why did CCT complex evolve eight different subunits? Perhaps, one possible explanation could be that certain specific combinations of subunits interact with specific structural features or motifs of the protein substrates and hence eight different subunits give large number of combinations to accommodate a broad variety of substrates for their folding [66, 67]. Furthermore, the presence of eight different subunits encoded by eight paralogous genes indicates that every subunit might have some specialized role in the overall functioning of CCT. Phylogenetic analysis of these eight subunits suggested that functional specialization of the individual subunits took place in the early phase of eukaryotic evolution and associated with its cellular functions and became essential for its survival [68, 69]. The electron microscopy and single particle analyses show that the domain structures of CCT are similar to those of GroEL and archaeal thermosome, and three domains (equatorial, apical, and intermediate domains) are present in a single subunit [68]. The mammalian subunits of CCT are designated as CCT*α*, CCT*β*, CCT*γ*, CCT*δ*, CCT*ε*, CCT*ζ*, CCT*η*, and CCT*θ* which are corresponding to Cct1p-Cct8p in yeast. Using biochemical and genetic approaches, it has been shown that CCT is a single heterooligomer of eight subunits and the arrangement of the subunits has a unique pattern as shown in Figure 1 [70, 71]. The subunits in a ring are arranged as Cct1p→Cct5p→Cct6p→Cct2p→Cct3p→Cct8p→Cct4p→Cct7p in a clockwise orientation. The arrangement of subunits in CCT ring also addressed by modeling using existing data, and four possible models have been proposed with clockwise and anticlockwise orientations; however, the relative positions of the subunits in a ring remain same [71–74]. The phasing between two rings of CCT has been addressed by three-dimensional reconstructions generated by electron microscopy and using monoclonal antibodies against Cct4p and Cct5p. It has been shown that interring communications take place through two different subunits in all the eight positions [75]. Moreover, the subunits associated with initiation and completion of the folding cycle cluster together in both inter- and intraring arrangements [75]. 

 However, recent cryo-EM analysis of closed CCT suggested new positions in a ring for three subunits-Cct1p, Cct5p, and Cct7p, and the subunits in a ring are arranged as Cct1p→Cct7p→Cct5p→Cct4p→Cct8p→Cct3p→Cct2p→Cct6p in a clockwise orientation [76]. This observation identifies the existence of two-fold axis between two rings. It reveals a unique pattern of interring arrangement that generates three heterotypic interring contacts (Cct3p-Cct4p, Cct2p-Cct5p and Cct6p-Cct7p) and two homotypic interring contacts (Cct1p-Cct1p and Cct8p-Cct8p). As the relative positions of other five subunits remain same in both the models and only three are having deviation in each of these models, fine-tuning of biochemical and EM analyses will be required to fix their positions. Recently, crystal structure analysis has been done for CCT purified from bovine testis along with its natural substrate, tubulin in the open conformation [77]. This structure showed that substrate interacts with CCT in the loops of apical and equatorial domains. The organization of ATP binding sites indicates that the substrate is stretched inside the cavity. Moreover, the fold of each domain (equatorial, intermediate and apical) of the eight subunits of CCT found to be similar to those of *α*- and *β*-subunit of thermosome. The thermosome equatorial domains can be superimposed on their CCT homologues, though small rearrangements in the orientation of helices and loops along the equatorial domain were observed. In contrast to the crystal structures of GroEL and thermosome, apical domains of CCT showed a wide range of conformations, both in the central cavity and in the aperture of the lid domains [42, 78, 79]. This supports the existence of sequential, hierarchical mechanism of conformational changes induced by ATP.

### 3.2. Nucleotide Binding to CCT and Protein Folding

The binding of ATP to the subunits of CCT and its hydrolysis is absolutely required for the CCT-mediated folding of newly synthesized polypeptide or misfolded protein substrates [58, 60, 80]. In the absence of ATP or in presence of ADP, CCT remains in an open conformation in which substrate binding sites in the apical domains are exposed and bind the substrates. Addition of ATP induces the conformational changes and subsequently the central cavity is closed by built-in lid structure to confine the protein substrate inside the cavity and provides a secluded environment for its folding. However, just addition of ATP to CCT does not promote the lid closure as shown using non-hydrolyzable ATP analogs [65, 81]. Using non-hydrolyzable ATP analog (AMP-PNP), it has been demonstrated that AMP-PNP-CCT binds to actin and tubulin on one CCT ring [66, 82]. On the other hand, when CCT is blocked in its ADP-P_i_ state, no CCT-protein target complex was formed. This suggests that binding sites in the apical domain of CCT will be available for target proteins in ATP-CCT state but not in ADP-P_i_-CCT state. Therefore, nucleotide exchange and hydrolysis might be working as a regulatory switch for the binding of target proteins to CCT [83].

### 3.3. Lid Structure of CCT

The bacterial chaperonin GroEL has a cofactor GroES which is used as a “lid” for the closure of central cavity essential for protein folding [84]. However, the eukaryotic cytosolic chaperonin CCT/TRiC does not have any homologue of GroES to cap the central cavity [17, 78]. The crystal structure of archaeal chaperonin thermosome has shown the presence of protrusions emerging from the apical domain and arranged in an iris-like *β* sheet which is responsible for closing the central cavity [78]. These apical protrusions are unique to group II chaperonins, like thermosome and CCT, and they have been proposed to have GroES-like activity and act as a built-in lid that might open and close in an ATP-dependent manner [17, 85]. Compelling evidence for the requirement of lid responsible for encapsulating the unfolded polypeptides comes from the observations that lidless chaperonins lose the ability to fold stringent substrates [65, 86, 87]. The question is how does this “built-in lid” functions for the closing and opening of the central cavity for substrate binding and releasing? Though there are striking similarities between group I and group II chaperonins, the lid closure mechanism seems to be quite different from each other. The binding of GroES to GroEL occurs upon ATP binding to equatorial domain of the GroEL subunits whereas the lid formation in eukaryotic and archaeal chaperonins is triggered by the transition state of ATP hydrolysis, suggesting nucleotide cycle dependent mechanistic difference of lid closure [88]. In one study, using the *Thermococcus* chaperonin, it has been suggested that ATP binding/hydrolysis causes independent conformational changes in the subunits. However, complete closure of the lid is induced and stabilized by the interactions of the helical protrusions of different subunits [89].

### 3.4. Mutations Affecting ATP Hydrolysis

**Figure 2:**
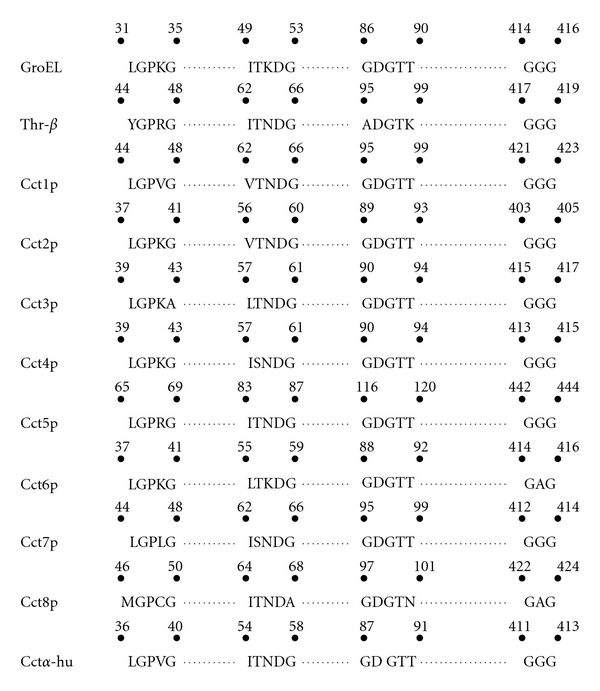
Comparison of highly conserved ATP binding/hydrolysis motifs in equatorial domain. GroEL, chaperonin of *E. coli*; Cct1p-Cct8p subunits of CCT complex of yeast *S. cerevisiae*; Cct*α*-hu, Cct1*α* subunit from human and Thr-*β*, *β* subunit from *Acidianus tengchongensis*. Starting from *E. coli* (homooligomeric chaperonin) to human (heterooligomeric chaperonin), all the chaperonin subunits have maintained conserved regions and any changes would have severe effects.

The high-resolution crystal structure of GroEL in ATP-bound and -unbound formed and that of GroES has identified an ATP binding domain encompassing N- and C-terminus of GroEL subunits [42, 90, 91]. This domain contains highly conserved GDGTT (residues 86–90) ATP binding residues along with loop structural motifs, LGPKG (residues 31–35), ITKDG (residues 49–53), and GGG (residues 414–416) which are found to be conserved among chaperonins. Sequence homology analysis of CCT subunits with that of GroEL identified almost identical ATP binding motif containing residues GDGTT and other loop structural motifs suggesting their conserved role in ATP binding/hydrolysis in prokaryotes as well in eukaryotes. Though the conserved loop elements appear to have certain common functions in ATP binding/hydrolysis, they could possess some functions which are specific to certain loop elements in a subunit. This is inferred from the observations that certain alleles of the same gene, affecting residues from different conserved loop structures have different degrees of cytoskeletal dysfunctions [92, 93]. Besides, there might be functional hierarchy among the paralogous motifs of different subunits. For example, homologous replacement of the fifth conserved glycine residue to glutamic acid in the LGPKG motif causes a lethal phenotype in Cct2p whereas the same mutation in Cct1p makes it heat sensitive in yeast *Saccharomyces cerevisiae*. However, the same replacement in Cct6p does not have much effect (Table 1 and Figure 2) [92, 94]. Furthermore, it has been shown that the conserved ATP binding/hydrolysis motif GDGTT→AAAAA replacement in the Cct6p in *S. cerevisiae *does not have much effect whereas same replacement is lethal for Cct1p [95]. From the above-mentioned experimental data, it can be suggested that different subunits play different role for ATP binding/hydrolysis in the CCT. This might be required for folding different substrates and modulating intraring and interring interactions. 

### 3.5. Substrate Recognition by CCT

One of the most well-studied chaperonins is the GroEL from *E. coli*, and the recognition of substrate by this chaperonin has been studied to a large extent, and so we will briefly discuss substrate recognition mechanism of GroEL before we go into the details of substrate recognition by CCT complex. A number of techniques have been used to unveil the mechanism of substrate binding by GroEL. The localization of substrate binding region appears to be at the entrance of central cavity of GroEL which has been shown by electron microscopy [34, 97]. The X-ray crystal structure revealed this substrate binding region in each subunit and has been termed “apical domain” [42]. A systematic mutational study was used to understand the role of different amino acids in this region, and it revealed that A152, Y199, S201, Y203, F204, L234, L237, L259, and V263 play important roles in binding the substrates as the mutations in these residues in the apical domain affect the substrates binding to GroEL severely [96]. Interestingly, most of these residues have hydrophobic side chains which can generate a hydrophobic surface for the binding of the substrate. On the other hand, mutations of charged amino acids in the apical domain appear to have no effect on the binding of the substrates. This suggests that hydrophobic residues in the apical domain are mainly responsible for creating hydrophobic surfaces in the central cavity for binding of substrates through hydrophobic interactions. However, the single-residue replacements in the intermediate domain (I150E, S151V, A152E, A383E, A405E, and A406E) exert global effect on the functioning of GroEL [96]. All of these mutants showed severe defect in ATPase activity though they fall outside the ATP-binding domain. Also the mutants at positions, 150, 151, 383, and 405 could bind polypeptide but the release of the polypeptide was severely affected. On the other hand, D87K/D87N mutation in the conserved domain, GDGTT, in the equatorial domain, lost the ATPase activity completely, though it has the ability to bind ATP. It has also reduced the ability to bind polypeptide; however, there was a complete block of polypeptide release [96]. 

Several techniques have been used to implicate the importance of hydrophobic interactions between GroEL surface and the substrates [98–102]. However, there are some exceptions to this substrate recognition principle and certain other forces such as electrostatic interactions might play an important role as well for binding the substrate to GroEL efficiently [103–105].

Although group I chaperonin (GroEL) and group II chaperonin (CCT) have double ring structure and share sequence similarities, they differ from each other in two major aspects. First, group I chaperonin is composed of identical subunits and has seven subunits per ring whereas group II chaperonin is composed of 2–8 paralogous subunits with 30–40% homology to one another and each ring has 8–9 subunits [16, 17, 26, 106, 107]. For example, CCT is composed of eight paralogous subunits [52, 70]. However, the functional relevance of this subunit diversity is not well understood. As the sequence divergence in the apical domain is more among the paralogous subunits, it has been hypothesized that different subunit in CCT has different substrate specificity [107, 108]. Second, group II chaperonins do not have GroES-like cofactor; however, it possesses a helical protrusion that acts as “built-in lid.” These two major differences might be related to the evolution of these group II chaperonins for assisting the folding of different archaeal and eukaryotic proteins. The structural and mechanistic differences between two groups might have profound functional impact on the substrate specificity [13, 109]. For example, several eukaryotic protein including actin and tubulin can be folded by CCT only whereas the bacterial proteins which require the assistance of GroEL for their folding, are not able to fold in eukaryotic cytosol [13, 58, 109, 110]. 

The specificity of GroEL and CCT towards the substrates is thought to be due to chemical nature of their interactions with substrates. It has been well established that GroEL recognizes the exposed hydrophobic surfaces of unfolded substrates [111–113]. On the other hand, CCT subunits possess specific binding sites for unique polar motifs of certain cellular proteins [82, 114–116]. However, using a biochemical approach, it has been shown that for the binding of actin, von Hippel-Lindau tumor suppressor and G*β* WD-40 protein to CCT, hydrophobic interactions are involved [117–119]. In the absence of well-defined structural surfaces or motifs present in the substrates of CCT, three model proteins, actin, tubulin, and von Hippel-Lindau tumor suppressor have been studied thoroughly to find the recognition sites present in these substrates as well as in the interacting subunits of CCT.

#### 3.5.1. Recognition Sites in Actin and Tubulin

Several studies have pointed out that the nature of actin and tubulin conformations bound to CCT are not of any nonspecific structures as in the case of GroEL substrates rather they must have some kind of defined, quasinative conformations before they are recognized by CCT for the final steps of their folding [80, 120–122]. The quasinative conformation may be achieved themselves or they may be guided by prefoldin kind of cochaperonin to reach that conformation. The atomic structure of actin has shown the presence of two domains, small and large [123]. The three-dimensional reconstruction analysis of alpha actin with CCT using electron microscopy has shown that CCT interacts with these two domains of actin by two specific and distinct interactions. The small domain of actin interacts with Cct4p subunit whereas large domain interacts with either Cct2p or Cct5p (both are 1,4 position with respect to Cct4p). This observation led to the suggestion that CCT interacts with actin in subunit-specific and geometry dependent-manner [115]. The three-dimensional reconstruction analysis combined with immunomicroscopy and screening study using actin peptide arrays have identified residues in four regions of actin molecule [114–117, 124]. Two of these regions ( R37-D51 and R62-T66) are located at the tip of small domain and interacts with Cct4p subunit of CCT. The other two regions (E195-R206 and T229-I250) are present at the tip of large domain and interact with Cct2p or Cct5p subunit. Mutational analysis coupled with electron microscopy and biochemical assay have shown that major determinants of actin binding to CCT are present at the tip of the large domain [116, 125]. A mutation (G150P) in the conserved putative hinge region between small and large domains resulted in the accumulation of actin on the chaperonin CCT. Furthermore, electroncmicroscopic studies have shown that actin interacts with Cct2p or Cct5p subunits rather than Cct4p subunit. It was thought that Cct2p and Cct5p might have highest substrate affinity, and this possibility has been strengthened by immunoprecipitation experiments of actin-CCT complexes [114]. 

On the other hand, the interaction of tubulin with CCT seems to be much more complex and it does not confine to a few regions of tubulin rather it is spread along its entire sequence and interacts with several domains at a time [82, 117, 122, 124, 126, 127]. Several studies have shown that CCT binding sites in tubulin are present in loops exposed to the surface of native protein [67, 82, 128, 129]. Of the eight binding sites present in tubulin, three are located at N-terminal domain and five are placed in C-terminal domain. The N-terminal binding sites are T33-A57, S126-Q133, and E160-R164. Immunomicroscopic experiments have shown that residues T33-A57 interact with Cct1p or Cct4p whereas the residues S126-Q133 and E160-R164 interact with Cct7p or Cct8p [82]. The interaction of C-terminal domain of tubulin with CCT is much more complex than that of N-terminal domain. Five putative segments present in the C-terminal domain responsible for interaction with CCT are T239-K254, P261-H266, S277-V288, V355-P359, and W407-E417, and they interact with multiple CCT subunits at a time. The segments T239-254 and P261-H266 interact with Cct6p or Cct3p subunits, the segments S277-V288 and V355-P359 interact with Cct5p or Cct2p whereas the segment W407-E417 interacts with Cct2p or Cct8p subunits [82]. 

Though it was suggested that tubulin binding sites could have weak interactions with CCT, the residues S277-V288 are thought to be hot spot for the binding of tubulin to CCT and it might have higher affinity to CCT compared to other sites [117, 122, 126, 127, 130]. Recently, Jayasinghe et al. used a computational approach to pinpoint the interactions between gamma subunit of CCT and its stringent substrate beta-tubulin. It has been shown that the substrate binding sites in CCT are composed of helical region (HL) and helical protrusion region (HP). Interaction of substrate at helical region involves both hydrophobic and electrostatic contacts while binding to helical protrusion is stabilized by salt bridge network [131].

#### 3.5.2. Recognition Sites in von Hippel-Lindau (VHL)

**Figure 3:**
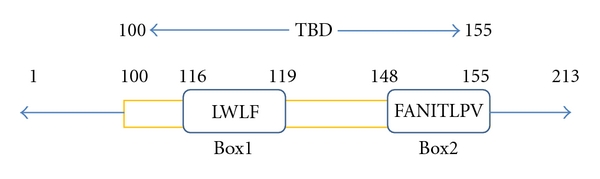
von Hippel-Lindau (VHL) protein containing two boxes, Box1 and Box2, required for binding to CCT. VHL is 213 amino acids long protein. Amino acid residues 100–155 constitute TRiC binding domain (TBD; this diagram was made on the basis of [118]).

The tumor suppressor protein von Hippel-Lindau (VHL) has been extensively studied from substrate point of view of CCT and has been shown to be an obligate substrate of CCT [14, 132]. VHL is a subunit of a ubiquitin ligase complex that targets cellular proteins, like HIF-1*α*, for proteolysis [133, 134]. Loss of function mutations in VHL is responsible for the tumor formation in kidney, adrenal glands, and central nervous system [135, 136]. VHL is composed of 213 amino acid residues of which 55-amino acid domain (100–155 residues) is necessary and sufficient for binding to CCT [132, 137]. Interestingly, most of the mutations in this domain are responsible for VHL diseases [138–140]. Using alanine-scanning mutagenesis procedure, the 55-amino acid segment has been completely analyzed to identify minimal regions responsible for CCT binding. This has revealed that two small regions of VHL, amino acid 116–119 (Box1) and 148–155 (Box2; Figure 3) are absolutely required for stable binding with CCT [118]. 

Furthermore, contribution of individual amino acid in these two boxes has been evaluated using single alanine substitution mutants. This analysis has shown that alanine replacement of W117 and L118 within Box1 or F148, I151, L153, or V155 within Box2 (Figure 3) substantially reduce the binding of VHL to CCT [118]. Though these two boxes are distant in the primary structure, they are located in the adjacent strands within the *β* sheet domain of folded VHL and the side chains of two boxes are projected in the same direction to generate a hydrophobic surfaces required for its interaction with CCT.

### 3.6. Allosteric Regulations in CCT

Allosteric regulation plays an important role for transitions between different functional states among the molecular machines in response to changes in environmental conditions. As it was strongly believed that allosteric regulation of chaperonins is crucial for assisting the protein folding, the chaperonins GroEL and CCT complex were studied from allosteric point of view to understand their functioning [47, 141]. The allosteric transitions of GroEL can be described by a nested allosteric model in which each of its rings is in equilibrium between a *T* state and an *R* state. The *T* state has low affinity for ATP and high affinity for unfolded substrate proteins whereas *R* state possesses high affinity for ATP and low affinity for unfolded substrates [142–144]. It has been shown that *T* and *R* states interconvert in a concerted manner in accordance with Monod-Wyman-Changeux (MWC) model of cooperativity [145]. However, in the presence of high concentration of ATP, GroEL ring switches from *TT* state to *RR* state via *TR* state in a sequential manner in accordance with Koshland-Némethy-Filmer (KNF) model of cooperativity [146]. Though the overall structure of both GroEL and CCT is similar, CCT is different from GroEL with respect to its subunit composition and so, it was important to unveil whether intra-ring transitions are concerted or sequential for CCT. Both biochemical and genetic approaches have been adopted to understand this mechanism. Kinetic studies have shown that CCT undergoes two ATP-dependent transitions and they most likely correspond to each of its two rings. ATP-induced conformational changes have been detected by monitoring changes in fluorescence and visualized using cryo-EM and single-particle reconstructions [66, 147–149]. It has been found that the binding of ATP to CCT generates an asymmetric particle in which one ring will have slight conformational changes whereas the other ring undergoes a substantial movement in the apical and equatorial domains [66]. Using the powerful yeast genetics, it has been suggested that intra-ring conformational changes in CCT are not concerted rather it occurs in a sequential manner around the ring. This was inferred from the suppression analysis of different mutant alleles of Cct1p, Cct2p, Cct3p, and Cct6p [70]. Moreover, EM analysis has shown two important differences between GroEL and CCT. First, a lot of conformational heterogeneity has been observed in the apo state of CCT but not in GroEL. Second, ATP-induced conformational changes take place in a sequential manner in CCT whereas concerted mechanism is observed for GroEL [150]. Biochemical as well as genetic analysis data suggested that ATP-induced conformational changes in CCT take place in the order Cct1p→Cct3p→Cct2p→Cct6p [70, 150].

### 3.7. Cochaperones of CCT

The role of CCT has been well established for the folding of a large number of proteins. However, it was not clear in the beginning whether CCT alone is sufficient for the folding of nascent chains to their maturity or other components are also required. Later, it was found in a genetic screen during the identification of synthetic lethals for gamma-tubulin that GimC, also known as prefoldin (PFD), participates in the maturation of cytoskeletal proteins [151]. Using biochemical approach, it has been shown that prefoldin plays an important role for the formation of functional actin and tubulin by transferring unfolded protein substrates to CCT [152]. The role of prefoldin (GimC) has been established in the folding of actin using chaperone trap and suggested that prefoldin acts along with CCT for the maturation of the substrate protein [153]. Using *in vitro* transcription/translation of actin, it has been shown that unfolded actin polypeptide chain remains bound to prefoldin until it is transferred to CCT. Similar observations were made for the maturation of *α*- and *β*-tubulin as well [154]. Prefoldin is a heterohexameric protein complex that exits both in archaea and eukaryotes. However, eukaryotic prefoldin is composed of six different subunits whereas archaeal prefoldin has only two different kinds of subunits: *α* and *β* subunits. It is possible that like CCT, eukaryotic prefoldin has been developed to more complex structure from simpler archaeal form to heterohexameric structure to participate in the more complex protein folding processes. Three-dimensional reconstruction of CCT with prefoldin based on electron microscopy analysis has shown that prefoldin interacts with each of CCT rings in a unique conformation with two specific subunits that are placed in a 1,4 arrangement. Therefore, it is highly desirable that PFD : actin complex will interact with CCT through Cct4 and Cct2 subunits or Cct4 and Cct5 subunits [74]. A large body of evidence show that heterohexameric complex of prefoldin uses its jellyfish or octopus-like structure to grip nonnative protein substrates and transfer it to CCT for proper folding [74, 155, 156].

Another set of proteins implicated in the regulation of CCT function are phosducin-like proteins (PhLPs) which were originally identified as modulators of heterotrimeric G protein signaling [157]. Subsequently, they were found to play an important role in the regulation of CCT function [158–162]. PhLPs are subdivided into three families like PhLP1, PhLP2, and PhLP3 and they share structural similarities at N-terminal helical domain, a central thioredoxin-like fold, and a C-terminal extension [163]. PhLP1 has been shown to have inhibitory effect on CCT and this may be required for regulating the protein folding capacity of CCT [158]. Electron microscopy reconstruction of mammalian CCT : PhLP1 has demonstrated that PhLP1 binds to apical domains of several chaperonin subunits [159]. Furthermore, the interaction of PhLP2 with CCT was suggested in proteome-wide studies. To substantiate this observation, *in vitro* study was done using human PhLP2A and has been shown that it does inhibit the folding of actin and forms ternary complex with CCT and actin in mammalian system [164]. However, recent study has suggested stimulatory role of PLP2 in yeast *S. cerevisiae* [165]. It has been shown that PLP2-CCT-ACT1 complexes produce 30-fold more actin than CCT-ACT1 complexes in a single ATP driven cycle. PLP2 itself can bind to actin through its C-terminal of thioredoxin fold and CCT-binding subdomain 4 of actin [165]. The inhibitory effect of human PDCL3, an orthologue of PLP2, can be relieved by exchanging the acidic C-terminus extension of that of PLP2 of yeast [165]. Therefore, it seems that higher eukaryotes have developed another level of regulatory control of CCT by phosducin-like proteins. The third member of this family, PhLP3, has been shown to bind CCT as well. The PhLP3 forms ternary complex with CCT and actin or tubulin, and does inhibit the folding process. It has been suggested that this negative impact is not due to direct competition for substrates rather by diminishing the ATPase activity of CCT by PhLP3 [162]. Moreover, *in vivo* experiments have shown that yeast PhLP3 might coordinate the proper biogenesis of actin and tubulin with prefoldin [162]. So, it is clearly established that phosducin-like proteins are responsible for regulating the function of CCT along with normal function of G protein signaling [161, 162, 164, 166]. Therefore, both prefoldin and phosducin-like proteins are working as co-chaperones to modulate the function of CCT. 

In another study, it has been shown that caveolin-1 can interact with CCT and modulates its protein folding activity [167]. The caveolin-1-TCP interaction involves the first 32 amino acids of the N-terminal segment of caveolin. Phosphorylation at tyrosine residue 14 of caveolin-1 induces the detachment of caveolin-1 from CCT and activates actin folding [167]. Recently, Bag3 protein has been identified as another co-chaperone of CCT. Bag3 protein is known as co-chaperone of Hsp70/Hsc70 and involved in the regulation of various cell processes, such as apoptosis, autophagy, and cell motility. Using RNAi, it has been shown that strains lacking Bag3 activity slowed down-cell migration and also influenced the availability of correctly folded monomeric actin [168]. Altogether, it shows that CCT is highly regulated by cochaperones for its folding activity of actin and other proteins and the interaction of different co-chaperones with CCT decides the fate of the final folding process.

### 3.8. Phosphorylation of CCT

**Figure 4:**
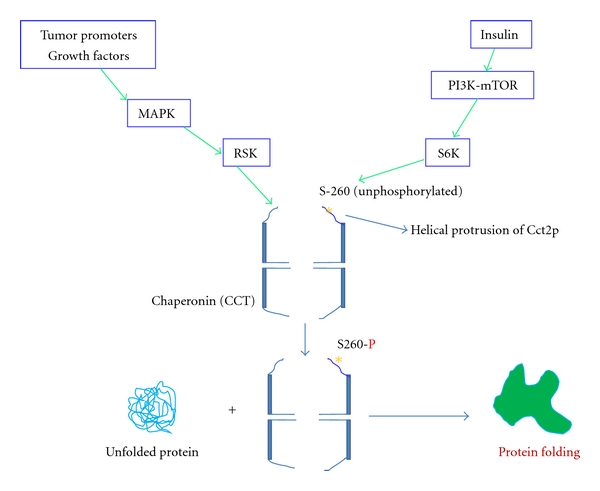
Schematic of Cct2p phosphorylation by p90 ribosomal S6 kinase (RSK) and p70 ribosomal S6 kinase (S6K) and the function of CCT containing phosphorylated Cct2p. *Indicates the S-260 position in the helical protrusion of Cct2p (this schematic is made based on the [169]).

Recently, it has been demonstrated that p90 ribosomal S6 kinase (RSK) and p70 ribosomal S6 kinase (S6K) can phosphorylate CCT in response to tumor promoters or growth factors that activate the Ras-mitogen activated protein kinase (MAPK) pathway [169]. RSK and S6K phosphorylate Ser-260 of Cct2p (Figure 4). Furthermore, it has been shown that Cct2p plays an important role in regulating cell proliferation and especially the phosphorylation of Cct2p at Ser-260 contributes substantially to this [169]. Though Cct2p has been implicated in this process, how the phosphorylation of Cct2p modulates the function of CCT is not clear. However, there could be two implications of this phosphorylation. First, phosphorylated Cct2p subunit itself might be interacting with certain factors of Ras-MAPK and PI3K-Mtor-pathways and regulate the cell proliferation in response to multiple agonists in diverse mammalian cells. Second, the phosphorylation of Cct2p subunit of CCT might change the folding rate and reduce the stress-related unfolded proteins in the cell. On the other hand, it has been shown that a fraction of GroEL is phosphorylated at least one phosphate at each of its subunits in *E. coli* [170, 171]. This phosphorylation of GroEL subunits enhances 50–100 -fold capacity of this chaperonin to bind to denatured proteins. Possibly, the phosphorylated form of GroEL might be responsible for refolding or degradation of certain damaged polypeptides [171]. In another study, it has been shown that GroEL of *Thiobacillus ferrooxidans* is phosphorylated in response to phosphate starvation suggesting its role in sensing and regulating stress responses in bacteria [172]. It is plausible that CCT carrying phosphorylated form of Cct2p might be playing similar roles in eukaryotes.

### 3.9. Cooperation of CCT with Upstream Chaperones

Many newly translated proteins may interact with several different chaperones before they reach their biologically functional three-dimensional structures. One of the most abundant molecular chaperones is Hsp70 which was found to associate with CCT *in vivo* suggesting their cooperation in protein folding [59, 132]. *In vitro* experiments were performed to elucidate the cooperative nature of Hsp70 and CCT using mammalian cell-free lysates. It has been shown that short chains of actin and firefly luciferase can interact with Hsp70 whereas longer ones interact with CCT [173, 174]. Besides, the cooperation between prefoldin (GimC) and CCT were found for folding of actin and tubulin [152–154]. Using three-dimensional reconstruction of CCT : PFD based on cryoelectron microscopy, it was shown that prefoldin binds to CCT through two specific subunits [74]. Moreover, several studies have also suggested the interaction between phosducin-like proteins and CCT indicating their cooperative nature for protein folding [158, 159, 165]. The interaction of caveolin-1 with CCT also modulates the protein folding function of CCT [167]. Furthermore, it has been shown that sequential cooperation between Hsp70 and Hsp90 plays an important role for the folding of steroid hormone receptors and kinases [175, 176]. 

From the above-mentioned experimental evidence, it appears that cooperation between different chaperones is a central principle to the protein folding process. However, cooperation may not be required for each newly synthesized polypeptide chain. For example, it has been shown that many chaperones are recruited to the protein synthesis machinery, and as soon as the polypeptide chains are coming out, they are protected by these chaperones including CCT. It has been demonstrated that chaperones can bind to ribosome-bound polypeptide chains in both prokaryotes and eukaryotes [173, 174, 177–183]. It seems that the cellular proteins follow different chaperone-dependent and chaperone-independent pathways for reaching their biologically functional three-dimensional structures. The proteins in the chaperone-independent pathway will probably be small proteins and they can fold without any help from any chaperones. The chaperone-dependent folding process might follow three different strategies depending upon the nature of the proteins. First, newly synthesized proteins will be bound by Hsp70, Hsp90, or other small chaperones transiently at the exposed hydrophobic regions present on the surface of nonnative proteins and will be protected from aggregation and will fold. Second, some proteins will be bound by Hsp70 and Hsp90 sequentially and will proceed for folding. Third, a small fraction of newly synthesized polypeptide chains will bind sequentially to Hsp70, Hsp90, and prefoldin/phosducin-like proteins and then transferred to CCT for final folding.

### 3.10. Protein Misfolding Diseases and CCT

The concerted and cooperative action of a large number of molecular chaperones leads to the production of three-dimensional and biologically functional protein molecules. The failure in this process may result in the aggregation and misfolding of many essential proteins and may cause a severe effect on the overall functions in a cell. Now it has been well recognized that protein aggregation and misfolding are the root causes for many diseases known as “protein misfolding” or “protein conformational” diseases [184]. The toxic effect of the aggregated or milfolded protein could be because of gain-of-function which will have titrating effect on interacting proteins or due to the loss of function of misfolded proteins [185, 186]. Aggregation process is a multistep process having stable or metastable intermediates that lead to the formation of homotypic fibrillar aggregates that may interact with other proteins and result in the formation of inclusion bodies or plaques that deposit outside or inside the cells [187–189]. A large body of evidence shows that the intermediates of the aggregate formation process are responsible for disease pathogenesis rather than the final products which may be inactive or protective [190, 191]. For example, increased level of diffuse polyglutamine- (polyQ-) expanded huntingtin is thought to be cause of cell death in Huntington's disease and inclusion bodies could enhance the survival [192]. Similarly, proteinaceous deposits or inclusion bodies are found to be not associated with toxicity in Parkinson's and Alzheimer's diseases [192–194]. 

Though chaperonins are generally responsible for maintaining the cellular protein homeostasis, they are now implicated in the pathogenesis of misfolding human diseases as well. Both the group I and group II chaperonins are found to be participating as modulators of misfolding diseases. For example, inactivation of mitochondrial Hsp60 is responsible for hereditary spastic paraplegia, a late-onset neurodegenerative disease [195, 196]. Recent studies have clearly shown that polyQ-expanded huntingtin is a potential substrate of CCT [197–199]. Moreover, an RNA interference screen in *C. elgans* has identified six out of eight subunits of CCT as suppressors of polyQ aggregation, suggesting that CCT can bind polyQ and inhibit the formation of toxic aggregates [200]. It has been shown that overexpression of Cct1p of CCT was effective at inhibiting huntingtin aggregation and subsequently increased the viability [198]. On the other hand, the deletion of Cct6p of CCT increases the huntingtin aggregation and toxicity [52, 199]. Generally, the CCT substrates are large, hydrophobic proteins containing the regions with *β*-strand propensity, and they are highly prone to the formation of toxic aggregates [63, 117, 118, 201]. Aggregation of these proteins may begin with conformational transition from native monomer to mature amyloid fibrils [197, 202, 203]. Therefore, it is quite possible that CCT binds directly to *β*-sheets and protects the protein from being aggregated which can be otherwise toxic for the cell. It has been shown that overexpression of certain subunits of CCT can protect from misfolding diseases. Therefore, drug-mediated induction of molecular chaperones can be considered as one of the methods for treating these diseases [50]. Otherwise, certain CCT subunits can be injected directly on regular basis like insulin in case of diabetic patients and misfolding diseases can be handled.

## 4. Conclusion

Molecular chaperones are crucial for the production of biologically functional three-dimensional protein structures. A large number of molecular chaperones are present in all the three kingdoms of life implying their importance in biological system. Chaperonins are cylindrical structures having central cavity for encapsulating unfolded protein substrates and assist in protein folding in an ATP-dependent manner. The chaperonin CCT is composed of eight different but related subunits of differential functional hierarchy in which catalytic cooperativity of ATP binding/hydrolysis takes place in a sequential manner rather than concerted cooperativity as found in GroEL. Moreover, substrate recognition in CCT takes place through diverse mechanisms involving hydrophobic and electrostatic interactions. For the fine-tuning of protein process, many cochaperones like prefoldin, phosducin-like proteins act as upstream factors and transfer the substrate to CCT. These upstream molecular chaperones and chaperonins might be responsible for generating a protective chaperone cage for the newly synthesized polypeptide chains to minimize the chance of aggregation and misfolding. Recently, it has been shown that certain CCT subunits are phosphorylated in response to tumor promoters or growth factors suggesting the possible roles of different kinases and possible certain phosphatases in regulating the activity of CCT. Any abnormal function posed by any chaperone at any stage of protein folding might have severe consequences. Many mutations in the molecular chaperones are now linked to Parkinson's and Alzheimer's diseases. Better understanding of chaperonin CCT and other molecular chaperones will be helpful to develop drugs for the treatment of misfolding or conformational diseases.

## Figures and Tables

**Table 1:** Mutations in the conserved ATP binding/hydrolysis domain and phenotypes in GroEL and three subunits of CCT complex.

Subunit	Amino acid replacement	Phenotype	References
GroEL	G35E	Normal	[96]
GroEL	D87N	Lethal	[96]
GroEL	D87K	Lethal	[96]
Cct1p	G45S	CS	[93]
Cct1p	G48E	HS	[92]
Cct1p	D96E	CS	[93]
Cct1p	G423D	HS	[93]
Cct2p	G41E	Lethal	[94]
Cct6p	G38S	Normal	[95]
Cct6p	G41E	TBZ^S^, NaCl^S^	[95]
Cct6p	G59R	TBZ^SS^, NaCl^S^	[95]
Cct6p	D89E	TBZ^S^	[95]
Cct6p	G90E	TBZ^SS^, NaCl^S^	[95]
Cct6p	G414E	CS	[95]
Cct6p	G416D	Normal	[95]
Cct6p	G416E	CS	[95]

CS: Cold sensitive; HS: heat sensitive; TBZ^S^: Thiabendazole sensitive; TBZ^SS^: Thiabendazole-hyper sensitive; NaCl^S^: Sodium chloride sensitive.
